# Recent Progress on Solid Substrates for Surface-Enhanced Raman Spectroscopy Analysis

**DOI:** 10.3390/bios12110941

**Published:** 2022-10-30

**Authors:** Kun Ge, Yuling Hu, Gongke Li

**Affiliations:** School of Chemistry, Sun Yat-sen University, Guangzhou 510006, China

**Keywords:** surface-enhanced Raman spectroscopy, solid substrate, construction, applications, perspectives

## Abstract

Surface-enhanced Raman spectroscopy (SERS) is a powerful vibrational spectroscopy technique with distinguished features of non-destructivity, ultra-sensitivity, rapidity, and fingerprint characteristics for analysis and sensors. The SERS signals are mainly dependent on the engineering of high-quality substrates. Recently, solid SERS substrates with diverse forms have been attracting increasing attention due to their promising features, including dense hot spot, high stability, controllable morphology, and convenient portability. Here, we comprehensively review the recent advances made in the field of solid SERS substrates, including their common fabrication methods, basic categories, main features, and representative applications, respectively. Firstly, the main categories of solid SERS substrates, mainly including membrane substrate, self-assembled substrate, chip substrate, magnetic solid substrate, and other solid substrate, are introduced in detail, as well as corresponding construction strategies and main features. Secondly, the typical applications of solid SERS substrates in bio-analysis, food safety analysis, environment analysis, and other analyses are briefly reviewed. Finally, the challenges and perspectives of solid SERS substrates, including analytical performance improvement and largescale production level enhancement, are proposed.

## 1. Introduction

Surface-enhanced Raman spectroscopy (SERS) has emerged as a powerful and useful vibrational spectroscopy analytical technique with unique superiorities in terms of non-destructivity, ultra-sensitivity, rapidity and fingerprint features [1]. With the rapid development of SERS substrates in recent years, SERS has achieved obvious achievements in several fields, including chemical [2], food [3], environment [4], and biological analysis [5]. In general, the original Raman signals of analytes are inherently weak on account of the smaller Raman scattering cross-sections, indicating the irreplaceable role of substrates in boosting Raman signals [6]. In general, the enhancement mechanisms, including electromagnetic (EM) and chemical (CM) enhancement, can be used to enlarge the Raman signals. For EM, when the frequency of incident is in resonance with the frequency of localized surface plasmon on the surface of metal, the incident electric field can be amplitude, as well as the Raman signals of molecules absorbed on the surface of metal [7]. For CM, the charge transfer (CT) between the substrates and molecules can enlarge the Raman cross-section of molecules, leading to higher Raman signals [8]. The noble metal nanoparticles (gold, silver, copper, etc.) are widely used due to their ideal SERS signal enhancement and simple preparation process [9]. However, the SERS performance of molecules, including sensitivity and stability induced by metallic colloids, mainly relies on the aggregation state of metal nanoparticles, this necessitating a gentle detection environment and improvement of molecule–metal interactions [10]. Therefore, several matrixes, including SiO_2_ or Al_2_O_3_ [11], polymers [12], metal-organic frameworks (MOFs) [13,14], semiconductor [15,16], and carbon materials [17,18], have been applied to combine with noble metal nanoparticles to improve the inherent instability of metallic colloids in SERS analysis. However, the development of high-quality substrates to generate sensitive and stable SERS signals can also be achieved through changing the form of substrate. In the past few years, more and more substrates with solid form, namely solid SERS substrates, have been developed due to their promising sensitivity, high stability, controllable morphology, and convenient portability. The fabrication of solid SERS substrates provides a promising strategy to improve the SERS performance in terms of stability, sensitivity, and portability.

Solid SERS substrates represent a special class of SERS substrate with solid features compared to colloid substrates. The general SERS measurements based on liquid substrates will meet limitations in terms of unstable signals, lower SERS activity, and short storage time. The solid SERS substrates show significant features in several aspects: (1) various preparation methods, including filtration, spin coating, chemical vapor deposition, magnetron sputtering, and self-assembly, are available for the fabrication of solid SERS substrates; (2) highly dense hot spots originating from concentrated unit area confirm the high SERS signals; (3) controllable micro morphology and structure, as well as good repeatability and reproducibility, provide uniform and stable SERS signals; (4) the solid features can significantly improve the stability of Raman signals to expand the scope of SERS analysis in practical applications, especially in reducing the requirement for sample pretreatment. Considering several superiorities of solid substrates in practical SERS analysis, the engineering of high-quality solid SERS substrates can significantly improve the analytical performance for SERS analysis.

**Figure 1:**
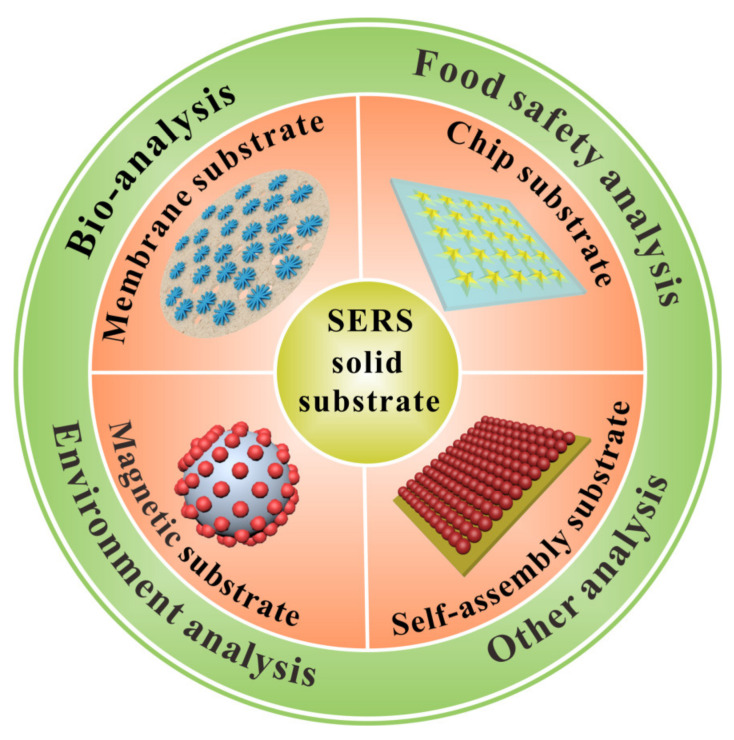
Various solid SERS substrates employed in analytical applications.

In this paper, we comprehensively summarize the recent advances made in the field of solid SERS substrates for SERS analysis in the last five years (2017–2022). The main content of this review can be briefly described in Figure 1. Firstly, the common construction methods for solid SERS substrates are briefly introduced. Secondly, the main categories of solid SERS substrates, including membrane substrate, self-assembled substrate, chip substrate, magnetic substrate, and other solid substrate, were reviewed systematically. Then, the representative analysis applications in several fields, such as bio-analysis, food safety analysis, environment analysis, and other analyses, are introduced. Finally, the challenges and perspectives of solid SERS substrates for SERS analysis, including analytical performance improvement and sample pretreatment capability enhancement, are proposed. We sincerely hope that this review can help and inspire more researchers in developing useful solid substrates for SERS analysis with high analytical performance.

## 2. Common Construction Methods

The solid SERS substrates can be fabricated by immobilization technologies, mainly including printing/writing, filtration, self-assembly, spin coating, magnetron sputtering, surface modification, deposition, in-situ grown, and immersion, etc. Printing/writing is a traditional technology with metallic colloids as inks, exhibiting unique features in terms of low cost, simplicity, and great potential for largescale production to support the fabrication of membrane/chip substrates [19,20]. However, the instability of metallic colloids over time and single material source limit their commercialization. Filtration has been widely used to fabrication of paper membrane substrates due to its low cost, simplicity, potentials in largescale production and abundant available materials [21,22]. In general, the materials can be trapped by cellulose-based paper matrix with unique spatial reticular structure under external forces. The stability of paper membrane substrates over time can be improved by using plasmon-free materials compared to printing substrates [23,24]. The construction of solid SERS substrates by self-assembly method with controllable morphology, uniform and dense distributions of “hot spot” can be realized under three-phase system (water-ethanol-cyclohexane/hexane/chloroform) combined with drying-mediated self-assembly mechanism [25,26,27]. In addition, some chemical inert template supports, including polystyrene spheres (PS), SiO_2_, and melamine-formaldehyde (MF), can significantly extend storage time compared to pure metallic colloids [28,29,30]. Spin coating is a simple and effective method for the fabrication of solid SERS substrates. The colloidal solution containing materials was sputtered onto the surface of solid supports at a certain speed, confirming the tight and uniform distribution of materials [31,32]. The various available materials and solid supports endow such materials with great potential in terms of commercialization for the fabrication of solid SERS substrates by the spin coating method. Magnetron sputtering is an effective method belonging to physical vapor deposition (PVD). The electrons will bombard targets under the interaction between magnetic field and electric field, resulting in the sputtering of targets on the surface of solid supports. The uniformity and thickness of targets can be controlled through sputtering voltage, pressure, and time [33,34]. The magnetron sputtering provides an efficient and mechanized fabrication method for solid SERS substrates, exhibiting great potential in terms of largescale production. However, the high cost of magnetron sputtering instruments and targets limits their commercialization process. Surface modification is a traditional fabrication method for solid SERS substrates, which is mainly dependent on the interactions, including electrostatic adsorption [35] and covalent bonds [36], between materials and solid supports. However, the disadvantages, including the long time required for surface modification and inhomogeneity of substrates, still need to be solved. The other fabrication methods, including deposition, in-situ growth, and immersion were seldom used, thus supporting the necessity of further development.

## 3. Categories of Solid SERS Substrates

Solid SERS substrates have drawn great attention for their features of solid characteristics, controllable morphology, and good stability compared traditional liquid substrates. Up to now, several solid SERS substrates, including membrane substrate, self-assembled substrate, chip-based substrate, magnetic substrate, and other solid substrates, have been fabricated for SERS analysis. Detailed information concerning representative solid SERS substrates, including categories, used materials, fabrication methods, analytical applications, and analytical performance, is listed in Table 1.

### 3.1. Membrane Substrate

The membrane substrate is one of the most common solid substrates for SERS analysis due to the easy preparation procedure, flexibility, satisfied enhanced performance, and good portability. Membrane substrates can be divided into paper membrane substrates, polymer membrane substrates, and non-support membrane substrates. The basic information, preparation procedure, and analytical performance of membrane substrates are introduced in detail.

#### 3.1.1. Paper Membrane Substrates

**Figure 2:**
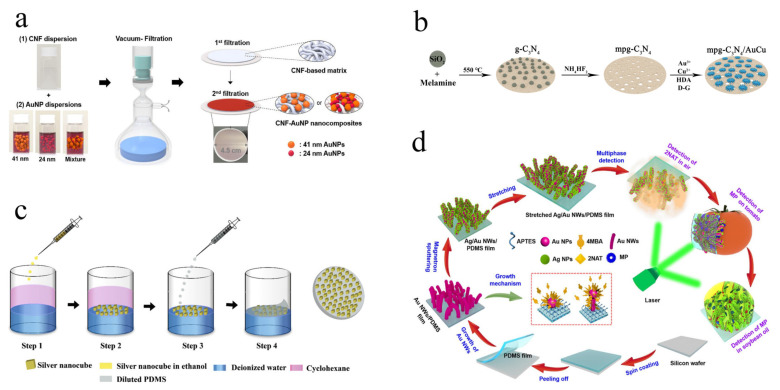
Schematic fabrication of CNF-AuNP membrane substrate (**a**). © 2022 Elsevier B.V. Adapted with permission from [42]. Schematic illustration of preparation principle of mpg-C_3_N_4_/AuCu membrane (**b**). © 2022 Elsevier B.V. Adapted with permission from [45]. Schematic illustration of fabrication process of Ag NCs@PDMS substrate (**c**). © 2022 American Chemical Society. Adapted with permission from [79]. Schematic preparation of Ag/Au NWs/PDMS film and its application for on-site detection of pesticides (**d**). © 2022 Elsevier B.V. Adapted with permission from [80].

The paper membrane substrates is a flexible solid SERS substrates based on the cellulose-based paper matrix with simple and convenient preparation process, including filtration [37], in-situ grown [38], printing/writing [39,40], self-assembly, and dropping/dipping [21,41]. Paper matrix shows promising spatial reticular structure for loading multifarious materials with high stability and capacity. To date, several materials have been used for the fabrication of paper membrane substrates, including noble metal [42], noble metals doped semiconductor composites [43], and noble metals embedded mesoporous material composite [44]. The dense distribution of “hot spots” originating from materials within limited space can significantly produce a good enhancement effect for SERS analysis. The noble metal is an ideal candidate due primarily to the simple preparation process and promising Raman signal enhancement. You’s group proposed a typical paper membrane substrate based on cellulose nanofiber-gold nanoparticles (CNF-AuNPs) composites via a simple filtration method (Figure 2a) [42]. The spatial distribution of AuNPs, including the number density and size ratio, can be precisely controlled, which can ensure the good enhancement signal due to the promising inter-particle plasmon coupling effects. The limit of detection (LOD) for rhodamine 6 G (R6G), thiram, and tricyclazole can reach 10.0 pmol/L, 1.0 pmol/L, and 10.0 pmol/L, respectively. This study provided a simple and novel strategy for improving SERS signals via controlling the spatial distribution of AuNPs on paper with nanoscale surface roughness via a simple and convenient preparation process. The noble metals doped semiconductor composite is an ideal SERS substrate due to the synergistic effect of localized surface plasmon resonance (LSPR) properties of noble metals and charge transfer of semiconductor. Our group [45] have developed a branched AuCu nanoalloy doped mesoporous graphitic carbon nitride hybrid hydrophobic membrane (mpg-C_3_N_4_/AuCu) for sensitive and selective analysis carcinogens including benzidine and zearalenone in food samples (Figure 2b). The high sensitivity was based on the design strategies of the anisotropic structure of AuCu, mesoporous semiconductor of mpg-C_3_N_4_, and hydrophobicity. The LOD for crystal violet (CV), benzidine and zearalenone were 1.0 ng/L, 0.14 μg/L and 0.03 μg/L, respectively. In addition, the stability and repeatability of mpg-C_3_N_4_/AuCu membrane substrate ensure the feasibility in practical SERS analysis. A black phosphorus-Au (BP-Au) filter paper membrane substrate was developed by Zhang’s group with low LOD of 1.0 nmol/L for CV molecule [43]. The paper membrane substrates based on noble metals doped semiconductor composite show superiorities in stability and SERS sensitivity compared to pure noble metals. The noble metals embedded mesoporous material composite has attracted increasing attentions due primarily to their significant preconcentrating and sieving effect. Recently, Au@ZIF-8 and UiO-66 (NH_2_)/AuNPs/Nylon-66 paper membrane substrates were respectively developed by Kang’s and Li’s group for trace analysis of contaminants in food samples [44,46]. The combination of mesoporous material with noble metals can selectively and effectively pre-concentrate targets in proximity to “hot spot” generated by noble metals according to the molecule size, suggesting the good SERS performance in sensitivity and selectivity. The paper membrane substrate shows great potential for largescale production due to the successful development of the multichannel filtering device and good serviceability for different types of materials.

#### 3.1.2. Polymer Membrane Substrate

The polymer membrane substrate is a typical flexible and transparent membrane substrate compared to conventional rigid and nontransparent substrates, providing a direct method for the analysis of molecules on irregular and nonplanar surfaces by simple covering [76]. Commonly used polymers are polydimethylsiloxane (PDMS) and poly (vinylidene fluoride) (PVDF) according to their promising thermodynamic properties, low cost, and ease of preparation [35,52]. In general, surface modification and assembly methods have been adopted for the fabrication of polymer membrane substrates [77,78]. Li et al. developed Ag nanocubes@PDMS film (Ag NCs@PDMS) via the assembly method for SERS analysis with high performance [79]. The dense Ag NCs arrays were formed at the cyclohexane/water interface via a three-phase system (water, ethanol, and cyclohexane), followed by the replacement of cyclohexane by PDMS mixture to form a flexible Ag NCs@PDMS film on the top (Figure 2c). The uniform and dense distribution of Ag NCs on PDMS matrix ensure the promising SERS signal enhancement, as well as good stability and flexibility for SERS analysis. The LOD for methylene blue (MB) and R6G can reach 0.1 and 1.0 μmol/L. A PDMS based membrane substrate modified with forest of Ag/Au nanowires (Ag/Au NWs/PDMS) was developed by Jiang’s group via surface modification for multiphase SERS analysis [80]. The separated PDMS film was first prepared by simple spin coating. Then, Au nanowires were in-situ grown on the PDMS film, followed by magnetron sputtering of Ag (Figure 2d). The integration of Au/Ag NWs and PDMS film provides a simple and novel strategy for the fabrication of a polymer membrane substrate with high SERS sensitivity (LOD = 1.0 μmol/L for 2-naphthalenethiol). In addition, some metal-semiconductor composites were immobilized on polymer membrane-based substrate for improving the SERS sensitivity and maintaining the stability, such as PDMS/TiO_2_/Ag [81], AgNW@rGO-PEI/PVDF-HFP [82], and Cu@rGO-PEI@AgNWs [83] membrane substrates. The polymer membrane substrate shows unique superiorities in accurate contact with samples, high SERS sensitivity, and easiness in tailoring. However, the polymer membrane substrate also meets limitations in short storage time and single enhanced material.

#### 3.1.3. Non-Support Membrane Substrate

**Figure 3:**
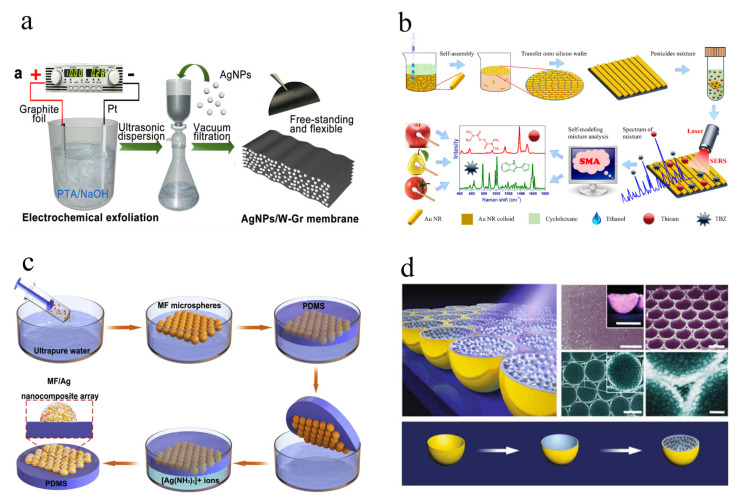
Schematic of the fabrication process of the AgNPs/W-Gr membrane (**a**). © 2022 Elsevier B.V. Adapted with permission from [24]. Schematic preparation of self-assembly gold nanorods (Au NRs) array and detection of pesticides on fruit surface (**b**). © 2022 Elsevier B.V. Adapted with permission from [87]. Schematic preparation diagram of hierarchical MF/Ag array substrate (**c**). © 2022 Elsevier B.V. Adapted with permission from [60]. Schematic illustration of preparation of NP-on-WS hierarchical substrate (**d**). © The Author(s) 2018. Adapted with permission from [61].

The non-support membrane substrate is a new kind membrane substrate without support, which was usually fabricated by a filtration process, with subsequent peeling from the supporter. The good membrane-forming property is mainly dependent on the mechanical and two dimensional (2D) properties of as-fabricated materials. Graphene (GO) based composite is promising in the fabrication of non-support membrane substrates on the basis of its good membrane-forming property and enhancement effect [84]. Ding’s group developed a non-support membrane based on multilayer graphene and AgNPs by cross-section strategy, maximizing the plasmonic coupling of AgNPs [24] (Figure 3a). The LOD for R6G can reach 0.5 pmol/L with long-term stability. The A multifunctional non-support membrane was prepared by intercalating gold nanoparticles (GNPs) and graphitic carbon nitride (g-C_3_N_4_) into graphene oxide nanosheets (GNPs/g-C_3_N_4_/GO) for SERS analysis of 4-chlorophenol at nanomolar level [55]. The sheet-like g-C_3_N_4_ and GO provide membrane-forming property, a loading site for GNPs, and CM effect all-in-one. Furthermore, Xi’s group developed several non-support membrane substrates based on pure transition metal oxides/nitrides, including N-TiO, γ-Mo_2_N, and δ-MoN, for high SERS analysis with good performance [23,56,85]. The high requirement for the fabrication of non-support membrane substrates indicates the great space for exploring new matrix material with good membrane-forming property. The non-support membrane substrates also show promising potential for commercialization in practical applications by virtue of their simple fabrication procedure and strong toughness.

### 3.2. Self-Assembled Substrate

Self-assembled substrates have widely adopted for SERS analysis according to their controllable morphology and dense distributions of “hot spots”. Liquid self-assembly substrates and solid self-assembly substrates are the main two important forms, usually fabricated by liquid metallic colloids and solid template array, respectively. Compared to membrane substrates, self-assembled substrates show unique advantages, including precise control of morphology, lateral spacing, and macroscale homogeneity [57]. The precise control of noble metals at nanoscale ensures the significant and stable SERS signals, exhibiting great potential for SERS analysis.

#### 3.2.1. Liquid Self-Assembly Substrate

Liquid self-assembly substrate with solid form mainly refers to the self-assembled metallic colloids transferred to the surface of the support film. The three-phase system (water-ethanol-cyclohexane/hexane/chloroform) is commonly applied for the fabrication of self-assembled metallic colloids interface under the guidance of drying-mediated self-assembly mechanism [27,86]. The metallic colloids can be driven to a limited area without a decrease in number, making significant and stable enhanced Raman signals due to the dense and uniform distribution of “hot spots”. Sun’s group fabricated an interfacial self-assembly gold nanorods (Au NRs) array for the simultaneous screening of thiram and thiabendazole (TBZ) in fruits with high sensitivity and reproducibility (Figure 3b) [87]. The LOD for thiram and TBZ are 36.0 pmol/L and 0.95 nmol/L with relative standard deviation (RSD) less than 10%. In addition, several self-assembly strategies have been adopted in the precise control of size, lateral spacing, morphology, and uniformity, such as organic solvent assist [25,57], convective self-assembly [26], electrostatic repulsion combined with depletion attraction [88], and ligand exchange [89], etc. The above self-assembly strategies show identical superiority in the self-assembly of anisotropic noble metals, which can significantly improve the SERS sensitivity on the basis of their denser “hot spot” and tuned surface plasmon resonance (SPRs) properties [90,91]. However, the instability of noble metals over time limits their practical applications in SERS analysis, needing to be solved or improved urgently.

#### 3.2.2. Solid Self-Assembly Substrate

Solid self-assembly substrates are composed of periodic solid array supports and noble metals, which have similar advantages to the liquid self-assembly substrate in satisfied and stable SERS signals thanks to the solid array supports and uniform distribution of “hot spots”. Furthermore, some chemically stable supports also provide a good protective effect for noble metals, which can significantly extend storage time and expand the scope of SERS analysis. In general, periodic template (PS, SiO_2_, and MF) mediated arrays are commonly used supports for the fabrication of solid self-assembly substrates [60,92,93]. The monolayer periodic templates are first assembled at the gas–liquid interface and then transferred to the solid film. After that, the noble metals can be deposited on the surface of monolayer periodic templates via several methods, including magnetron sputtering [30], in-situ grown [60], surface modification [93], and deposition [28]. Deng et al. developed a hierarchical superhydrophobic melamine-formaldehyde/Ag (MF/Ag) array for the SERS analysis of biomolecules with good sensitivity and reproducibility (Figure 3c) [60]. The LOD of MF/Ag array for dopamine, lysozyme, and hemoglobin is 2.69 fmol/L, 62.0 pmol/L and 6.83 pmol/L, respectively. The good repeatability of the MF/Ag array was confirmed in several situations, including random sites, consecutive days, extreme temperature, and UV exposure. In addition, the periodic templates can also be designed and fabricated with different morphology, size, and spacing for more sensitive and stable SERS signals [29,92,94]. Apart from the direct modification of noble metal on templates, the solid templates can be removed to obtain vacancy with controllable morphology before noble metal modification [95,96,97,98]. A solid self-assembly substrate based on warped spaces to manipulate hotspots was developed by Chen’s group (Figure 3d) [61]. The Au nanobowl (Au NB) was firstly obtained by the template metal deposition method combined with etching method, followed by e-beam evaporation of thin SiO_2_ film. Finally, nanoparticles on a warped substrate (NP-on-WS) can be achieved by the gas-phase deposition method with AgNPs. The warped spaces of NP-on-WS substrate can significantly enhance broadband electromagnetic energy within large spectral regions, inducing promising SERS signals for R6G. In addition, some materials, including copper oxide (CuO) [99], graphene [100], and functional self-assembled monolayer [101], were applied to the fabrication of solid self-assembly substrates to improve the sensitivity, stability, and selectivity of the SERS method. In terms of practical SERS analysis, solid self-assembly substrates show better potential for commercialization compared to liquid self-assembly substrates on account of their stability, several available materials, and ease of modification. However, the fabrication method for solid self-assembly substrates needs to be simplified to meet the requirements for practical application.

### 3.3. Chip Substrate

**Figure 4:**
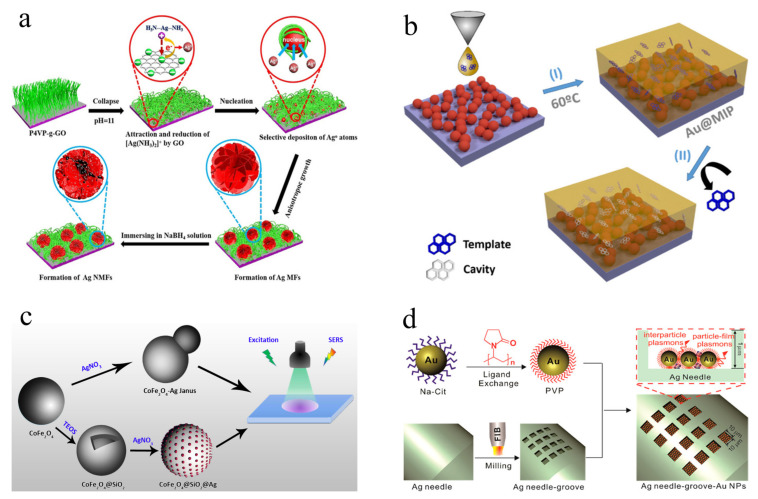
Schematic procedure for fabricating of 3D Ag MFs and Ag NMFs on P4VP-g-GO films (**a**). © 2022 Wiley-VCH Verlag GmbH & Co. KGaA. Adapted with permission from [64]. Schematic illustration of preparation of Au@MIP film (**b**). © 2022 American Chemical Society. Adapted with permission from [31]. Schematic synthesis of CoFe_2_O_4_/Ag substrate (**c**). © 2022 American Chemical Society. Adapted with permission from [107]. Schematic synthesis of Au-G-AgNs (**d**). © 2022 American Chemical Society. Adapted with permission from [74].

The chip-based substrate is an important supplement to the solid self-assembly substrate, especially during the preparation process. The fabrication of a solid self-assembly substrate is mainly based on periodic templates combined with the self-assembly strategy. However, chip-based substrates show more strategies, including in-situ grown [102], spin coating [36], electrochemical deposition [103], surface modification [104], and magnetron sputtering [33], etc. Thus, the structure of chip-based substrates can be designed and fabricated to meet the requirements of SERS analysis in terms of sensitivity, selectivity, and stability. A novel 3D Ag mesoflowers (Ag MFs) modified poly(4-vinylpyridine) brush-grafted-graphene oxide (P4VP-g-GO) films was developed by Chen’s group (Figure 4a) [64]. The obtained Ag nanoporous mesoflowers show large specific surface area and highly rough surface, resulting in ultra-sensitivity for the SERS analysis of 4-aminothiophenol with LOD of 0.1 pmol/L. Furthermore, the chip-based substrate with high selectivity for SERS analysis can be achieved via combination with the molecular imprinting technique (MIP) [105]. The MIP based method shows advantages in selectivity, versatility, and predictability, suggesting good feasibility for specific recognition in SERS analysis. Pastoriza-Santos’s group [31] has reported a reliable and selective molecularly imprinted chip-based SERS substrate (Au@MIP) for the highly selective analysis of polycyclic aromatic hydrocarbons (PAHs), including pyrene and fluoranthene (Figure 4b). The AuNPs were first deposited on the glass by the layer-by-layer method and then coated with thin MIP film for the fabrication of an Au@MIP chip substrate. The good SERS activity and selectivity of the developed method based on the Au@MIP chip substrate can be obtained through a short distance between SERS active regions and specific sites of molecules. Moreover, some chip-based substrates with satisfactory stability have been designed and fabricated. Yim’s group [106] developed a two-dimensional (2D) monolayer/Ag nanoparticle (AgNP) substrate with long-term oxidative stability. The AgNPs were assembled on the (3-aminopropyl) trimethoxysilane (APTMS) modified Si substrate, then covered by monolayer graphene or hexagonal boron nitride (h-BN). The hybrid platform of h-BN/AgNPs exhibited long-term SERS stability over 80 days, which can be attributed to the high chemical stability of monolayer h-BN. In addition, the h-BN/AgNPs substrate showed better stability compared to the graphene/AgNPs based substrate due to their different electrical and chemical properties. The chip-based substrates provide an effective way of selecting an appropriate preparation strategy according to requirement of practical SERS analysis, which is more likely to be commercialized.

### 3.4. Magnetic Substrate

Magnetic substrates represent a class of solid substrate with unique features of magnetic properties and SERS activity, which can realize sample pretreatment and in-situ SERS analysis all-in-one. In addition, the promising magnetic separation properties of magnetic substrate endows them with solid-like characteristic for SERS analysis. In general, the magnetic solid substrate shows the typical structure of a plasmonic substrate over a magnetic core (P-o-M) [70]. Wei et al. have reported two magnetic solid SERS substrates with P-o-M structure, including CoFe_2_O_4_@SiO_2_@Ag and CoFe_2_O_4_-Ag Janus (Figure 4c), which show good SERS activity due to the strong SPR effect of AgNPs and special micro-nano structure [107]. Furthermore, the developed two magnetic solid substrates also exhibit a stable SERS signal at about 90% after five repeated uses. In addition, the SERS performance of magnetic solid substrates can also be improved by several strategies. Two ternary magnetic MOF-based substrates of Fe_3_O_4_@Au@MIL-100 (Fe) [108] and CoFe_2_O_4_@ HNTs/AuNPs [69] with high SERS activity have been developed, benefitting from the good adsorption property of MOFs and HNTs, respectively. A Fe_3_O_4_@TiO_2_@Ag [51] magnetic substrate has been fabricated to improve SERS activity by using the synergistic effect of semiconductor characteristics of TiO_2_ and SPR properties of Ag. Several selective reagents have been used to improve the selectivity of the SERS method based on magnetic solid substrate, such as aptamer [71], antibody [109], cyclodextrin [68], and molecular imprinted polymer [110], etc. It should be noted that the stability and repeatability of SERS signal induced by magnetic solid substrates still meet limitations in terms of oxidation of the magnetic core and uneven distribution of plasmonic nanoparticles. Thus, the development of the SERS method based on magnetic solid substrate with promising stability as well as good sensitivity should be focused on in the future.

### 3.5. Other Solid Substrate

Apart from above four types of solid SERS substrates, there are some other solid SERS substrates that have been developed without specific classifications. Jun et al. [111] developed a hydrophobic sponge substrate via the deposition of silver nanowires coated h-BN composites on melamine-sponge (h-BN/AgNWs/sponge). The hydrophobic sponge substrate shows two important roles in the separation of organic pollutants via hydrophobic treatment and π–π interactions, as well as in-situ monitoring the absorption process of organic pollutants by the SERS method. An Au nanoparticles-decorated silver needle (Au-G-AgNs) was prepared by Zhou’s group for the ultrasensitive SERS analysis of malachite green (MG) in aquaculture products with low LOD of 0.1 nmol/L (Figure 4d) [74]. The surfaces of AgNs were milled to a groove array (10 × 10 μm) by focused ion-beam-scanning electron microscopy instrument equipped with a gallium-ion beam, followed by surface encapsulation of polyvinylpyrrolidone (PVP) modified AuNPs. The groove arrays not only provide reproducible SERS signals, but also avoid erasing AuNPs during insertion process. In addition, AgNPs decorated eggshell membrane [112] and adhesive tape-based substrate [113] were also reported for SERS analysis with high performance. These solid SERS substrates open a new vision for the fabrication of SERS substrate, as well as provide more choices for practical SERS analysis.

## 4. The Applications of Solid SERS Substrates

In view of the unique advantages of solid SERS substrates, several practical applications in fields of bio-analysis, food safety analysis, environment analysis, and biomedicine/public safety analysis have been reported. Here, we briefly introduce the specific examples of solid SERS substrates in fields mentioned above.

### 4.1. Bio-Analysis

**Figure 5:**
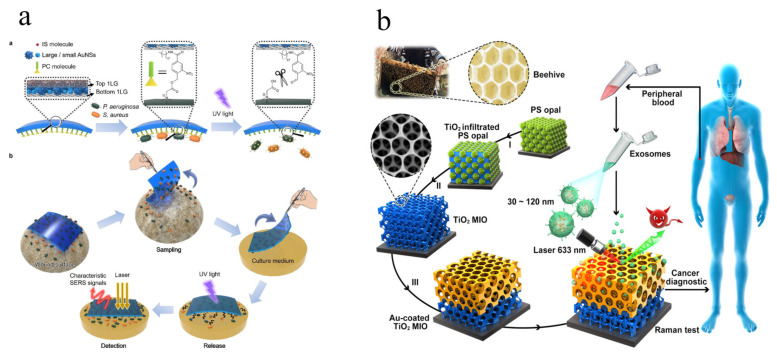
Schematic fabrication of adhesive tape based solid platform and its applications in rapid sampling, photocontrolled release, and on-site SERS detection (**a**). © 2022 American Chemical Society. Adapted with permission from [53]. Schematic preparation of Au-coated TiO_2_ MIO and its detection process (**b**). © 2022 American Chemical Society. Adapted with permission from [62].

With the rapid development of society and economy, the requirements for human health becoming higher and higher. Thus, development of high-quality solid SERS substrate for bio-analysis has attracted increasing attention [54,114]. The solid SERS substrates have been used for pathogen analysis with single molecule sensitivity and promising stability. Guo et al. developed an adhesive tape based solid platform for the analysis of wound infectious pathogens with the special advantage of three-in-one, namely rapid sampling, photocontrolled release, and SERS detection [53]. The dense distributions of gold nanostars were sandwiched between two layers of monolayer graphene modified by selective regent (*o*-nitrobenzyl derivative) for wound infectious pathogens (Figure 5a). Hence, the sensitive, stable, and selective SERS analysis of Pseudomonas aeruginosa (P. aeruginosa) and Staphylococcus aureus (S. aureus) could be achieved. The developed “three-in-one” platform opened up new horizons in applications of point-of-care (POC) by the SERS method with satisfactory analytical performance. Tian et al. [59] reported self-assembled plasmonic nanoarrays for the simultaneous and sensitive reproducible analysis of various bacteria with concentrations in the range of 50–2000 CFU/mL. The self-assembled GNPs show dense and uniform “hot spots”, making stable and reproducible SERS signals for the analysis of eight bacteria. Moreover, Zhao et al. [115] fabricated a magnetic aptamer-conjugated Fe_3_O_4_@Au (Fe_3_O_4_@Au-Apt) substrate for the selective and sensitive SERS analysis of S. aureus. The LOD for S. aureus can reach 25 CFU/mL with cell capture efficiency (CCE) of 68%. This multifunctional magnetic solid substrate integrated the advantages of specific recognition, sensitive SERS detection, and photothermal therapy (PTT) of S. aureus.

Apart from pathogens, several biomarkers of disease have been accurately determined with the SERS method by solid SERS substrate. Dong et al. [62] developed a beehive-inspired macroporous solid SERS substrate for the label-free analysis of the protein phosphorylation status of exosomes (biomarker of cancer) (Figure 5b). The multiple layers of PS opal templates were spin-coated on the Si wafer, followed by TiO_2_ precursor infiltration into the interstitials. After annealing, PS templates can be removed, and the TiO_2_ macroporous inverse opal (MIO) structure can be obtained. Finally, the MIO structure TiO_2_ framework was covered by thin Au film to form a beehive-inspired macroporous solid SERS substrate. The exosomes from plasma can be effectively captured and analyzed due to the macroporous structure, which was applied to identify cancer patients successfully by recording the SERS intensity of phosphoproteins (P-O bond) at 1087 cm^−1^. Kim et al. [28] developed gold-decorated hexagonal-close-packed polystyrene (Au/HCP-PS) chip-based substrate for the noninvasive and real-time analysis of biomarkers of asymptomatic breast cancer in human tears. The fabricated Au/HCP-PS arrays used for SERS analysis show ultra-sensitivity at femtomole-scale and good reproducibility (with RSD less than 5%) thanks to the periodic distribution of template and uniform distribution of AuNPs, respectively. A graphene oxide-gold nanostar (GO-GNS) hybrid membrane substrate was fabricated by Pan’s group for the label-free and rapid analysis of serum bilirubin (biomarker of jaundice) [21]. The LOD of the developed method based on GO-GNS hybrid membrane substrate for serum bilirubin can reach 0.436 μmol/L, attributed to the integration of enrichment effect and chemical enhancement of GO, and the dense “hot spot” of GNS. Li’s group developed a polytetrafluoroethylene (PTFE)-based membrane substrate for the sensitive detection of atherosclerosis-related biomarkers (cytokines), including IL-10 and MCP-1 [65]. A sandwich-like membrane substrate was fabricated by antibody modified AuNPs deposited nanoporous networking PTFE membrane for SERS analysis with high sensitivity (0.1 pg/mL for IL-10 and MCP-1) and specificity. The solid SERS substrates provide great potential in the label-free, rapid, sensitive, and on-site analysis of biomarkers of diseases, which will play a more important role in clinical diagnosis and treatment.

In addition, solid SERS substrates have also been applied to the analysis of some biomolecules with promising performance. Cai et al. [35] developed a flexible polymer membrane substrate of multibranched AuNPs embedded in polydimethylsiloxane (M-AuNPs-PDMS) for the accurate and direct analysis of hematin in erythrocytes without a separation process. The LOD for hematin can reach 0.03 nmol/L, which can be attributed to the synergistic effect of close contact between M-AuNPs and hematin, and plasmon coupling between the M-AuNPs and the Au film. A chip-based substrate of GO-supported L-cysteine-functionalized starlike AuNPs (L-cysteine SAuNPs@GO) was reported by Panikar et al. [36] for the analysis of paclitaxel and cyclophosphamide in serum samples. The space between the SAuNPs can be precisely controlled to 2.28 nm, confirming great SERS signals. In addition, L-cysteines play an important role in the brush layer to avoid the blocking of “hot spot” and fouling by proteins in serum. The LOD for paclitaxel and cyclophosphamide are 15.0 μmol/L and 5.0 μmol/L with high reproducibility. The SERS method based on solid substrates will play a vital role in the analysis of biomolecules with unique features of label-free, on-site, sensitivity, and stability.

### 4.2. Food Safety Analysis

In recent years, food safety issues have attracted increasing attention due to their close relationship with public health. Several food contaminants, including mycotoxins [116], pesticides [27], and food additives [117], show features of low concentration and high toxicity, demanding an appropriate analytical method to meet the requirements of trace analysis. The solid substrate is a promising candidate for SERS application with dense “hot spot”, high stability, controllable morphology, and convenient portability. Here, we briefly introduce the specific analysis applications for food contaminants based on solid SERS substrate.

**Figure 6:**
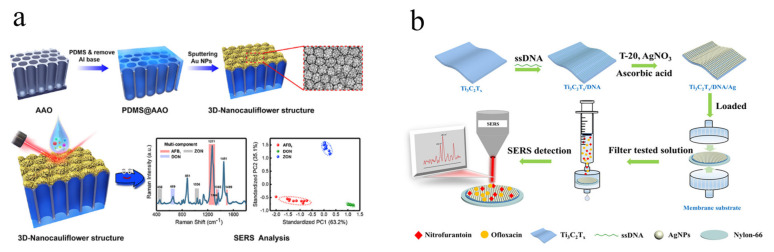
Schematic fabrication of cauliflower-inspired chip-based SERS substrate for simultaneous label-free analysis of multiple mycotoxins (**a**). © 2022 American Chemical Society. Adapted with permission from [33]. Schematic preparation process of the Ti_3_C_2_T_x_/DNA/Ag membrane substrate and its application of simultaneous and accurate quantification of multiple antibiotics (**b**). © 2022 American Chemical Society. Adapted with permission from [49].

Mycotoxins are class of important biotoxins in foods with widespread distribution, high toxicity, and thermal stability, indicating great potential threat to human health via several routes [118,119]. Considering the unique features of SERS method, solid SERS substrates can be used for analysis of mycotoxins with high analytical performance. Li et al. [33] fabricated cauliflower-inspired chip-based SERS substrate for simultaneous and label-free analysis of multiple mycotoxins in maize including aflatoxin B1 (AFB_1_), deoxynivalenol (DON), and zearalenone (ZEN) (Figure 6a). The LOD for 4-mercaptobenzoic acid (4-MBA), AFB1, DON, and ZEN are 0.15 pg/mL, 1.8, 47.7, and 24.8 ng/mL, respectively. Furthermore, the RSD of as-fabricated chip substrate for random 40 sites detection of 4-MBA was 4.57%, exhibiting excellent repeatability for SERS analysis. Shao et al. [120] developed a magnetic solid substrate based on Au(core)@Au-Ag(shell) modified Fe_3_O_4_ (Au@AuAg-MNP) for highly sensitive and selective detection of ochratoxin A (OTA) in red wine. The magnetic solid features combined with aptamer endowed with promising repeatability and selectivity by SERS method. Rostami et al. [121] fabricated Ag-capped silicon nanopillar array combined with supported liquid membrane (SLM) extraction for label-free and sensitive analysis of OTA in wine samples. The detection system achieves integrations of enrichment, high throughput and on-site detection, which has successfully applied to trace SERS analysis of OTA in wine samples with high sensitivity (LOD = 113 μg/L). Considering the wide distribution and high toxicity of mycotoxins, solid SERS substrates shows great potentials in on-site, high throughput, sensitive and stable detection of mycotoxins.

Pesticides are major pollutant sources of food, especially in vegetables and fruits, needing a stable SERS substrate for rapid, sensitive and trace analysis. Ma et al. [77] fabricated a flexible and transparent gold nanobush-PDMS hybrid membrane substrate (AuNB-PDMS) for analysis of TBZ in cherry samples. The grown AuNB on PDMS provides uniform and dense distribution of “hot spot”, indicating the sensitive and stable SERS signals. The flexible and transparent membrane substrate can realize in-situ and swabbing detection of TBZ with LOD of 0.64 ng/mL, which is suitable in on-site monitoring pollution status of TBZ. Wang et al. [122] developed a superhydrophobic membrane substrate for trace analysis of nitenpyram (NIT) based on silver dendrite-decorated filter paper. The superhydrophobic treatment can avoid randomly spreading of analytes, ensuring efficient sample collection. Additionally, anisotropic structure of fern-like silver dendrites provide uniform and strong electromagnetic field to enhance SERS signals and the LOD of NIT can reach 1.0 nmol/L. Xie et al. [123] fabricated a flexible membrane substrate with multiple “hot spots” based on three-dimensional (3D) AuNPs modified PDMS (Au@PDMS) for highly sensitive SERS analysis of pesticide residues. The membrane substrate shows typical features of flexibility and portability, exhibiting great potential in rapid and on-site detection of thiram on the nonplanar surface of vegetables and fruits. The flexible membrane substrates are more suitable for on-site, rapid and sensitive SERS analysis of pesticides according to their promising portability and deformability.

Food additives are important class of additives added to foods in order to significantly improve the quality or shelf life of food. However, several food safety issues caused by abuse or illegal addition of food additives remind that development of effective analytical method for monitoring food additives in food is still urgently required. Li’s group reported a Ti_3_C_2_T_x_/DNA/Ag membrane substrate for simultaneous analysis of multiple antibiotics including nitrofurantoin (NFT) and ofloxacin (OFX) in aquatic samples (Figure 6b) [49]. The good sensitivity of SERS method based on Ti_3_C_2_T_x_/DNA/Ag membrane substrate can be attributed to the excellent electromagnetic enhancement (EM) combined with chemical enhancement (CM). In addition, the promising uniformity, reproducibility, stability of as-fabricated membrane substrate was verified by several characterizations. The Ti_3_C_2_T_x_/DNA/Ag membrane substrate integrated a series of features of multitarget separation, enrichment, and in-situ detection. Luo et al. [124] developed flexible Ag nanowires embedded PDMS (Ag NWs@PDMS) membrane substrate for detection of MG residue in food samples. The as-fabricated Ag NWs@PDMS film shows great durability and recyclability in several situations including 20 times stretchability, HNO_3_ corrosion, ultrasonication and broad temperature in range of −20–80 °C. The sensitivity of developed SERS method based on Ag NWs@PDMS film was proved by quantitative detection MG in juices with LOD of 10.0 nmol/L. Kumar et al. [40] developed paper-based substrate by step-by-step printing method for sensitive and reproducible SERS analysis of non-permitted colourants including Metanil Yellow (MY) and MG. The Ag nanowires were in-situ grown on the printed area with high density, indicating the high sensitivity and reproducibility for SERS detection. Nie et al. [97] fabricated bowl-like pore array belong to solid self-assembly substrate for SERS detection of melamine in milk powder. The periodic hollow Au/Ag alloy array exhibits great SERS activity for melamine with LOD of 1.0 μmol/L, which meets the requirement of trace analysis of melamine in milk powder. The solid SERS substrates will play an important role in monitoring the abuse or illegal addition for food additives.

### 4.3. Environment Analysis

With the rapid development of economy and industry, environmental pollutants become more and more serious in diversities, wide sources, and high toxicity. Thus, developing advanced solid substrate for rapid, on-site, and sensitive SERS analysis of environmental pollutants is a good choice. Organic dyes are class of widespread pollutants, which have several sources including soil, water source, plants, and aquatic product, etc. Ji et al. [125] designed a chip-based substrate with sandwich structure of Ag/Cu_2_O/ITO by electrochemical deposition combined with in-situ reduction process for ultrasensitive and recyclable detection of RhB. The uniform distribution of Cu_2_O shows two main roles in protecting AgNPs and providing CM effect. The Ag/Cu_2_O/ITO integrated sensitivity for SERS analysis (LOD of 1.0 pmol/L for RhB), long-term stability (≥58 days), uniformity (RSD of 5.8% for 10 random points), and recyclablility (≥10 times), which exhibits great potentials in on-site monitoring of organic dyes. Das et al. [38] developed a large-scale paper-based membrane substrate for highly sensitive analysis R6G and RhB with LOD of 1.0 pmol/L and 1.0 μmol/L, respectively. In addition, the as-fabricated membrane substrate shows satisfied reproducibility for SERS analysis of R6G and RhB in rain, pond, and tap water. A surface molecularly-imprinted magnetic solid substrate (Fe_3_O_4_@MIP) was constructed for trace analysis of MG in tap water and carp samples with low LOD of 1.50 and 1.62 pmol/L, respectively [126]. Combining magnetic core with molecularly imprinted layer endow effective and selective separation and enrichment of MG in complex samples. Considering the uniform distribution of magnetic solid substrate, the SERS signal of MG can be significantly enhanced after the transfer of Fe_3_O_4_@MIP on AgNPs deposited Si wafer.

Apart from organic dyes, there are several environmental pollutants have been detected successfully by solid substrate, such as PAHs [127], atmospheric aerosol [95,128], pesticide residues, drug residues [52,129,130], etc. The structure of solid SERS substrate can be designed and fabricated to meet requirement of SERS detection of environmental pollutants with high analytical performance. However, there are few environmental pollutants categories have been reported for SERS analysis based on solid substrate. Thus, the practical analysis of environmental pollutants based on solid SERS substrate still exhibits great development space, which need further exploring in the future.

### 4.4. Other Analysis

The solid SERS substrates also have been applied to medicine and public safety analysis due to their portability, rapidity, sensitivity, and stability. White’s [131] group developed a AgNPs printed paper membrane substrate for monitoring of therapeutic drug (flucytosine) with LOD of 10.0 μg/mL. The developed membrane substrate combined with vertical-flow method can meet the requirement of rapid, on-site, and sensitive detection of flucytosine. Gupta et al. [132] fabricated a transparent and elastomeric film based on patched Au nanorods (AuNRs) modified PDMS for vapor-phase detection of explosives of trinitrotoluene (TNT). The as-fabricated film can realize the formation of dense hot spot by TNT capture process, indicating the large SERS enhancement. A solid substrate of AgNPs immobilized polyurethane sponge (PU@PDA@Ag) was constructed for trace SERS analysis of inorganic explosives including ClO^4−^, ClO^3−^, and NO^3−^ [133]. The swabbing extraction process significantly improve the collection efficiency and SERS performance. The LODs for ClO^4−^, ClO^3−^, and NO^3−^ were 0.012, 0.025 and 0.025 μmol/L with high reproducible, stable and uniform SERS signals. The solid SERS substrate will play an important role in on-site, rapid and sensitive monitoring the illegal addition of drugs and illegal abuse of explosives.

## 5. Challenges and Perspectives

During past few years, it witnessed the flourishing development of solid SERS substrate in fabricating advanced substrate and broadening the scope of applications. Solid SERS substrates exhibit superiority in improvement of SERS analytical performance by dense hot spot, high stability, controllable morphology, and convenient portability. Furthermore, solid SERS substrate still shows great potentials in mass production and commercialization. However, despite above proposed advantages and superiorities, the solid SERS substrate also meets some challenges, mainly are improving the SERS analytical performance and enhancing the mass manufacture level.

The SERS analytical performance is the key parameters for analytical applications, which is mainly dependent on design and fabrication of solid SERS substrate. The analytical performance solid SERS substrate still need improved in four main aspects. First, how to precise control nanogap of plasmonic nanomaterials within few nanometers on the whole plane under the guidance of target size, which is the key to produce sensitive and uniform enhanced signals with high selectivity. Second, many reported solid SERS substrate meet limitations in terms of anti-interference ability for real samples, especially for pure plasmon based solid substrates. Third, the storage time of some solid SERS substrates still needs to be improved for SERS analysis, such as the paper-based membrane substrate, chip-based substrate, and magnetic solid substrate. Fourth, the scope of applications of solid SERS substrates is mainly in bio-analysis and food safety analysis, which should be expanded to more fields. In addition, the single molecule detection technology in nanocavities based on solid SERS substrates has drawn great attention due to its ultrahigh sensitivity, especially for nucleic acid detection [134,135,136,137,138]. However, the single molecule detection for other analytical fields remains in the initial stage, showing great prospects for development. Finally, choosing proper solid substrates for analytical applications (bio-analysis, food safety analysis and environment analysis) still meets limitations in diversification, demanding improvements to identify the best solid substrate for analytical applications. For example, the biocompatibility of solid SERS substrates for bio-analysis is still necessary.

At present, the fabrication of solid SERS substrates remains at the laboratory level, which limits their development in commercial applications. Thus, how to achieve the integration of batch production, long storage time, and commercialization all-in-one is significantly important for realizing practical applications. Hence, designing and fabricating a solid SERS substrate with excellent analytical performance combined with batch potential should be further investigated.

An ideal solid SERS substrate shows unique features in terms of promising enhancement effect, uniform and stable SERS signals, long storage time, and simple preparation process compared to common liquid SERS substrates. Converting from the traditional liquid SERS substrate to a novel solid SERS substrate provides a great opportunity to improve SERS performance and develop an effective commercialization platform. The solid SERS substrate will be a powerful tool for SERS analysis after addressing the challenges mentioned above.

## Figures and Tables

**Table 1:** Summary of representative solid SERS substrates.

Classification	Materials Used	Fabrication Method	Stability/Reproducibility	Targets	LOD	Samples	Refs.
Paper membrane substrates	Ag/sandpaper/filter paper	Deposition	RSD of 12.9% for 1600 points	Ferritin antigen	31.6 pg/L	Water	[47]
	mpg-C_3_N_4_/AuCu	Filtration	RSD of 14 consecutive days (4.8%) and 25 batches (2.7%)	Benzidine	0.14 μg/L	Tea bag	[45]
				Zearalenone	0.03 μg/L	Food sample	
	GO-Au	Immersing	/	Bilirubin	7.0 μmol/L	Blood serum	[48]
	GO-GNS	Filtration	reproducibility of 3.6-8.9%	Bilirubin	0.44 μmol/L	Blood serum	[21]
	Ti_3_C_2_T_x_/DNA/Ag	Filtration	RSD of 4.1% for 42 days	Nitrofurantoin	12.0 μg/L	Fish samples Shrimp samples	[49]
				Ofloxacin	35.0 μg/L		
Polymer membrane substrates	PDMS/Au	Surface growth	/	*V. parahaemolyticus*	12 cfu/mL	Oyster, salmon	[50]
	Au NWs/PDMS	In-situ grown	mapping RSD values of 6.45% and 6.31% for PSA and AFP	Prostatespecific antigen α-fetoprotein	0.49 ng/L 0.72 ng/L	Serum	[51]
	PVDF/Ag/MIP	Mixed growth	/	Enrofloxacin hydrochloride	0.1 μmol/L	Water	[52]
	PMMA/GO/AuNSs	Immersing Spin coating	RSD less than 10.7% for SERS mapping; RSD of 4.7% for 600s irradiation	*P. aeruginosa* *S. aureus*	/	Skin burn wound	[53]
	PDMS/Ag	Surface growth	RSD of 5.6% for 6 random points	2,6-pyridinedicarboxylic acid	1.2 pmol/L	*B. subtilis*	[54]
Non-support membrane substrates	GNPs/g-C_3_N_4_/GO	Filtration	RSD of 14.1% 16 points within 4 batches	R6G	0.05 pmol/L	Water	[55]
	δ-MoN	Filtration	RSD less than 7.8% for 5000 measurement points	2,5-dichlorophenol BPA	0.1 nmol/L	Water	[56]
Liquid self-assembly substrates	AuNRs	Self-assembly	RSD of 12.9% for 10 reuse	R6G	1.0 nmol/L	Water	[25]
	Au@Ag	Self-assembly	RSD of 10.5% for substrate-to-substrate	Thiram	1.1 μg/L	Water	[27]
				Thiabendazole	51.0 μg/L		
	AuNRs	Solvent-assisted self-assembly	/	Pyocyanin	1.0 pmol/L	*P. aeruginosa*	[57]
	Au core/Ag shell nanostar	Self-assembly	RSD of 14.9% for 10 random points; 23 days for storage	Dopamine	0.1 nmol/L	Water	[58]
	AuNPs	Self-assembly	RSD of 2.7% for SERS mapping; RSD of 2.7% for 10 batches	*E. coli* *S. aureus*	50 cfu/mL	Serum	[59]
Solid self-assembly substrates	MF/Ag	Self-assembly; In-situ grown	Good repeatability and reproducibility for different random sites, consecutive days, extreme temperature, and UV exposure	Dopamine Lysozyme Hemoglobin	2.69 fmol/L 62.0 pmol/L 6.83 pmol/L	Water	[60]
	Au NB	Template deposition; Etching; In-situ grown	/	R6G	1.0 pmol/L	Water	[61]
	Au/TiO_2_	Template method; Sputtering	/	Exosomes	/	Plasma	[62]
	Au/HCP-PS	Spin coating; Deposition	RSD less than 5% for reliability and reproducibility	Tyrosine Phenylalanine Tryptophan	/	Tears	[28]
	AuNPs	Plasms etching Sputtering	/	alpha-fetoprotein- L3	3.0 ng/L	Water	[29]
	AuNPs, AgNPs	Etching Deposition	RSD of 11.6% for 100 random points; RSD of 12% for 2000 spectra within 4 batches	Pyocyanin	6.25 μmol/L	Artificial sputum	[63]
Chip-based substrate	P4VP-g-GO/Ag	Surface modification; In-situ grown	/	4-aminothiophenol	0.1 pmol/L	Water	[64]
	Au@MIP	Surface modification; Spin coating	RSD of 10.0–15.0% for SERS mapping	Pyrene	1.0 nmol/L	Creek water; Seawater	[31]
				Fluoranthene			
	M-AuNP/PDMS/Au	Surface modification	RSD of 3.8% for 10 random points	Hematin	0.03 nmol/L	Human erythrocytes	[35]
	SAuNPs@GO	Surface modification	/	Paclitaxel Cyclophosphamide	15.0 nmol/L 5.0 nmol/L	Blood serum	[36]
	AuNPs	Surface modification	/	MCP-1 IL-10	0.1 ng/L	Human serum	[65]
	AAO/Au NBPs	Diffusion	RSD of 9.4% for 5 random points; RSD of 9.6% for 5 batches	Dopamine	6.5 nmol/L	Human serum	[66]
	AuNPs/rGO/NFs	Surface reduction Surface growth	/	6-mercaptopurine	0.5 nmol/L	Human plasma	[67]
Magnetic substrates	Fe_3_O_4_@Au	Surface modification	RSD of 7.6% for 30 random points	Valosin-containing protein	25.0 ng/L	Human tissues	[68]
	CoFe_2_O_4_@HNTs/AuNPs	Surface modification	RSD less than 10% for reproductive determination	4,4′-thioaniline Nitrofurantoin	26.0 μg/L 14.0 μg/L	Hair dyes	[69]
						Fish feed; Aquatic samples	
	Fe_2_O_3_@AgNPs	Thermal decomposition	/	*P. gingivalis* *A. actinomycetemcomitans*	/	Human saliva	[70]
	Fe_3_O_4_@SiO_2_@Au	Surface modification	RSD less than 11.0% for reproductive determination	*S. aureus* *A. baumannii* *K. pneumoniae*	14.1 fmol/L 9.7 fmol/L 8.1 fmol/L	MDR bacteria-infected mice	[71]
	Fe_3_O_4_@Ag	Surface modification	RSD of 3.7% and 5.8% for 30 consecutive test in water and human serum	miRNA-122 miRNA-223 miRNA-21	349 amol/L 374 amol/L 311 amol/L	Human serum	[72]
Other solid substrate	hBN/AgNWs/sponge	Mixed modification	RSD of 8.0% for 30 random points	RhB	10.0 nmol/L	Water	[73]
	Au-G-AgNs	Surface modification	RSD of 9.2% for 30 random points	MG	0.1 nmol/L	Water	[74]
	Ag-cotton	Surface modification Tabletting	RSD of 7.5% for 10 random points	*E. coli*	/	Urine	[75]

## Data Availability

The data presented in this study are available on request from the corresponding author.
